# TTF-1 and p40 co-expressing non-small cell lung cancer with ERBB2 and TP53 gene mutations: A case report and review of the literature

**DOI:** 10.1097/MD.0000000000041290

**Published:** 2025-02-07

**Authors:** Jiachun Sun, Haolin Shi, Bo Sun, Tingting Wei, Jingxiang Su, Hongyan Liu, Dengkui Wang, Xinyang Li

**Affiliations:** a Henan Key Laboratory of Cancer Epigenetics, Cancer Institute, The First Affiliated Hospital, College of Clinical Medicine, Medical College of Henan University of Science and Technology, Luoyang, China; b School of Basic Medical Sciences, Henan University of Science and Technology, Luoyang, China.

**Keywords:** non-small cell lung cancer, prognosis, TTF-1/p40 co-expression

## Abstract

**Rationale:**

Non-small cell lung cancer (NSCLC) that co-expresses thyroid transcription factor-1 (TTF-1) and p40 represents a distinct subtype of lung cancer. This case report details a 53-year-old male patient with NSCLC exhibiting co-expression of TTF-1 and p40, highlighting the need for further investigation into this unique presentation of NSCLC.

**Patient concerns:**

A 53-year-old male, nonsmoker, presented to our hospital in November 2023 with complaints of cough, expectoration, chest pain, and dyspnea.

**Diagnoses:**

Computed tomography scans revealed perihilar lesions in the upper lobe of the right lung with distal obstructive pneumonia, invasion into adjacent mediastinal structures, multiple pulmonary metastases, possible mediastinal and right hilar lymph node metastases, pleural effusion, and a slightly thickened pleura in the right lung, along with multiple metastatic lesions in the liver. Immunohistochemical analysis showed positive staining for CK (AE1/AE3), TTF-1, and p40, while CK56, CD68, CD45 (LCA), D2-40, and Ki-67 (50%+) were negative, leading to the diagnosis of lung adenocarcinoma. Second-generation sequencing identified mutations in ERBB2 (EXON20.p.Y772-A775dup) and TP53 (EXON5.c.404G-Tp.C135F).

**Interventions:**

The patient underwent 6 cycles of chemotherapy with a regimen consisting of bevacizumab, pemetrexed, and carboplatin.

**Outcomes:**

Posttreatment evaluations on December 28, 2023, and February 25, 2024, indicated stable disease. The patient is currently on a maintenance chemotherapy regimen of bevacizumab and pemetrexed, and his condition is stable.

**Lessons:**

The co-expression of TTF-1 and p40 in NSCLC may define a novel subtype of lung cancer. Further research is imperative to elucidate its characteristics, molecular mechanisms, and to establish its morphological features and therapeutic strategies.

## 1. Introduction

Based on the latest statistical data, in 2022, 2.5 million new lung cancer cases and 1.8 million cancer-related deaths were recorded worldwide. Among all malignant tumors, lung cancer has the highest incidence and mortality rates.^[1]^ At present, histopathological examination is still the gold standard for lung cancer diagnosis. According to the World Health Organization (WHO) classification, lung cancer can be divided into 2 main categories: non-small-cell lung cancer (NSCLC) and small-cell lung cancer. Based on immunohistochemical features, NSCLC is classified into the following pathological subtypes: adenocarcinoma, squamous cell carcinoma, adenosquamous carcinoma, sarcomatoid carcinoma and large-cell carcinoma.^[2]^ Thyroid transcription factor-1 (TTF-1) and p40 are the most specific indicators used to distinguish adenocarcinoma from squamous cell carcinoma in the lung.^[3]^ Adenosquamous carcinoma can be identified based on the expression of TTF-1 and p40 by different tumor cell populations. However, there are only a few reports on NSCLC, which is a special type of lung cancer in which the same tumor cell population diffusely expresses both TTF-1 and p40.^[4]^ This study reported a case of poorly differentiated NSCLC with the diffuse co-expression of TTF-1 and p40 based on second-generation sequencing. Further, it retrospectively analyzed previous literature to explore the clinicopathological features, immunophenotypes, molecular features, diagnosis, differential diagnosis and treatment of NSCLC, thereby providing data that can improve its diagnosis and treatment.

## 2. Case description

**Figure 1.**
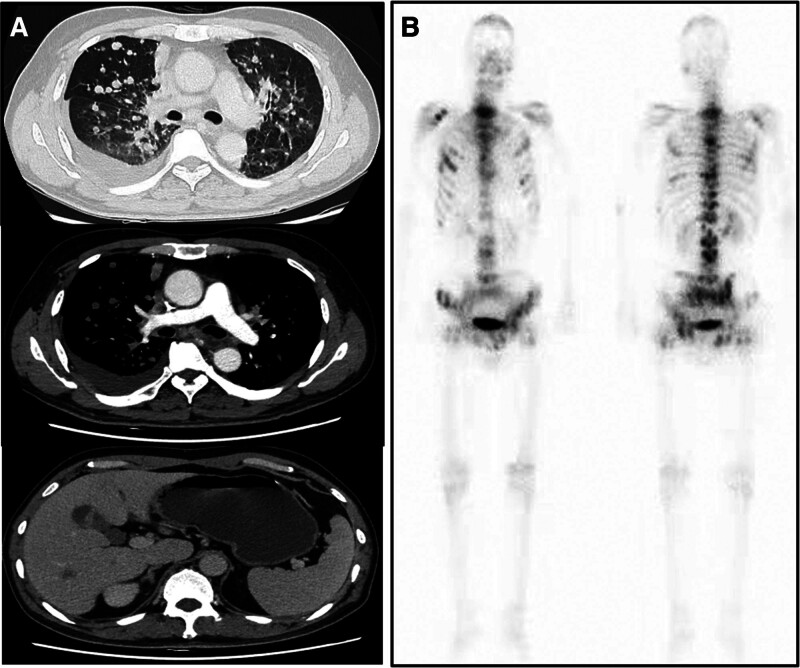
Image examination results. (A) CT showed perihilar lesions in the upper lobe of the right lung with distal obstructive pneumonia, invasion into the adjacent structures of the mediastinum, multiple metastases in the right and left lungs, multiple metastatic lesions in the liver. (B) Bone imaging revealed abnormal increases in the radioactivity of the skull, sternum, cervical, thoracic, lumbar and sacral vertebral bodies, multiple ribs on both sides, scapula, pelvis and lower limbs. CT = computed tomography.

**Figure 2.**
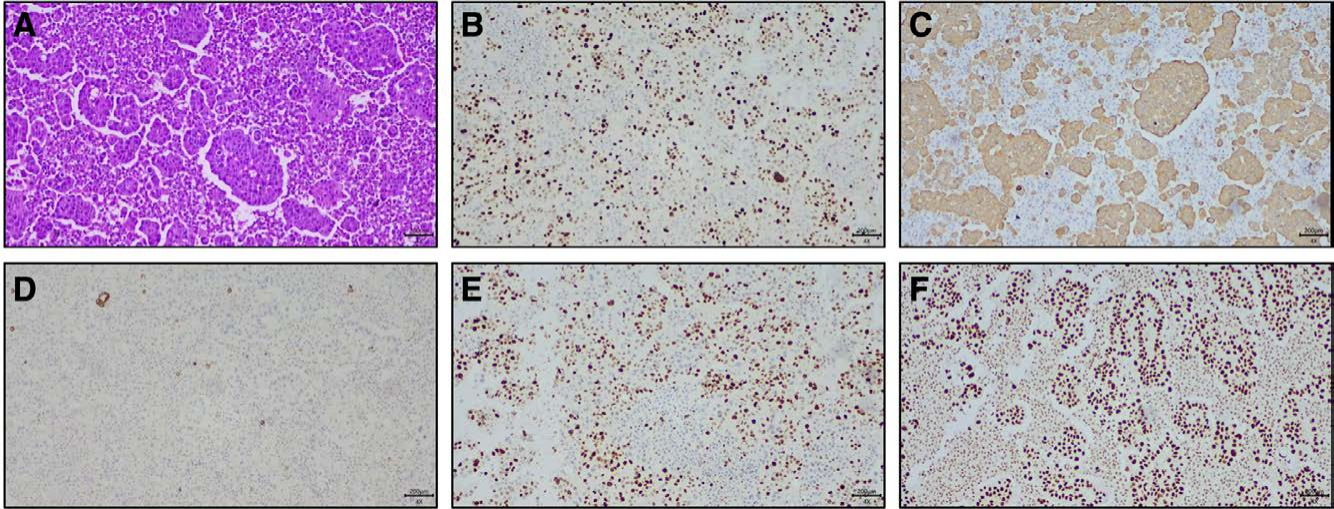
Immunohistochemical results of the cells stripped by drainage fluid. (A–F) Immunohistochemistry results, respectively, HE, Ki-67 (50%+), CK (AE1/AE3) (+), CK5/6 (‐), p40 (+), and TTF-1.

**Figure 3.**
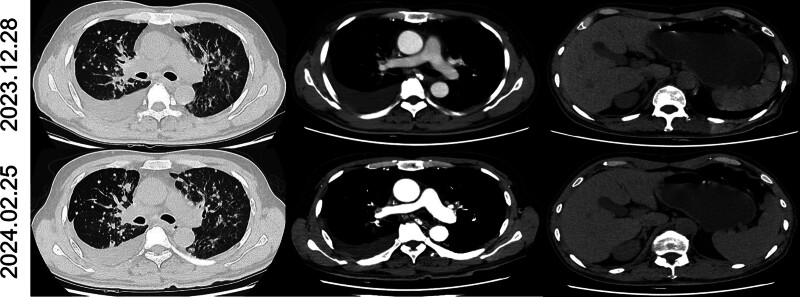
Second-generation sequencing. ERBB2 (EXON20.p.Y772-A775dup) and TP53 (EXON5.c.404G-Tp.C135F) mutations.

**Figure 4.**
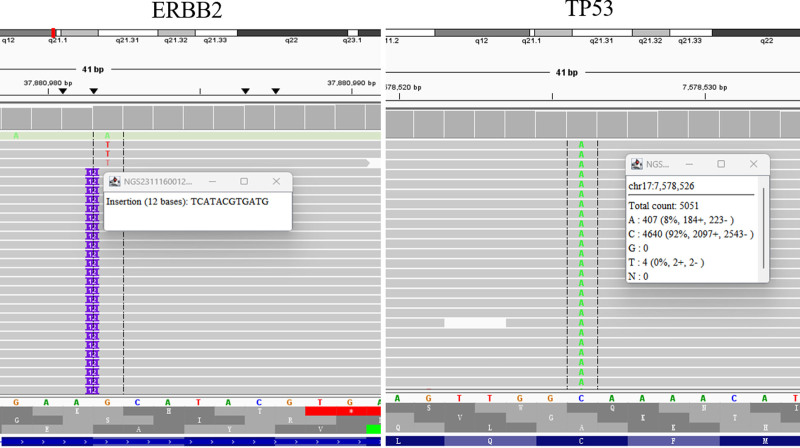
Response evaluation. The soft tissue shadow near the pulmonary portal in the upper lobe of the right lung decreased, the right pleural effusion had no significant changes, and the metastatic lesions in the liver had no significant change.

**Figure 5.**
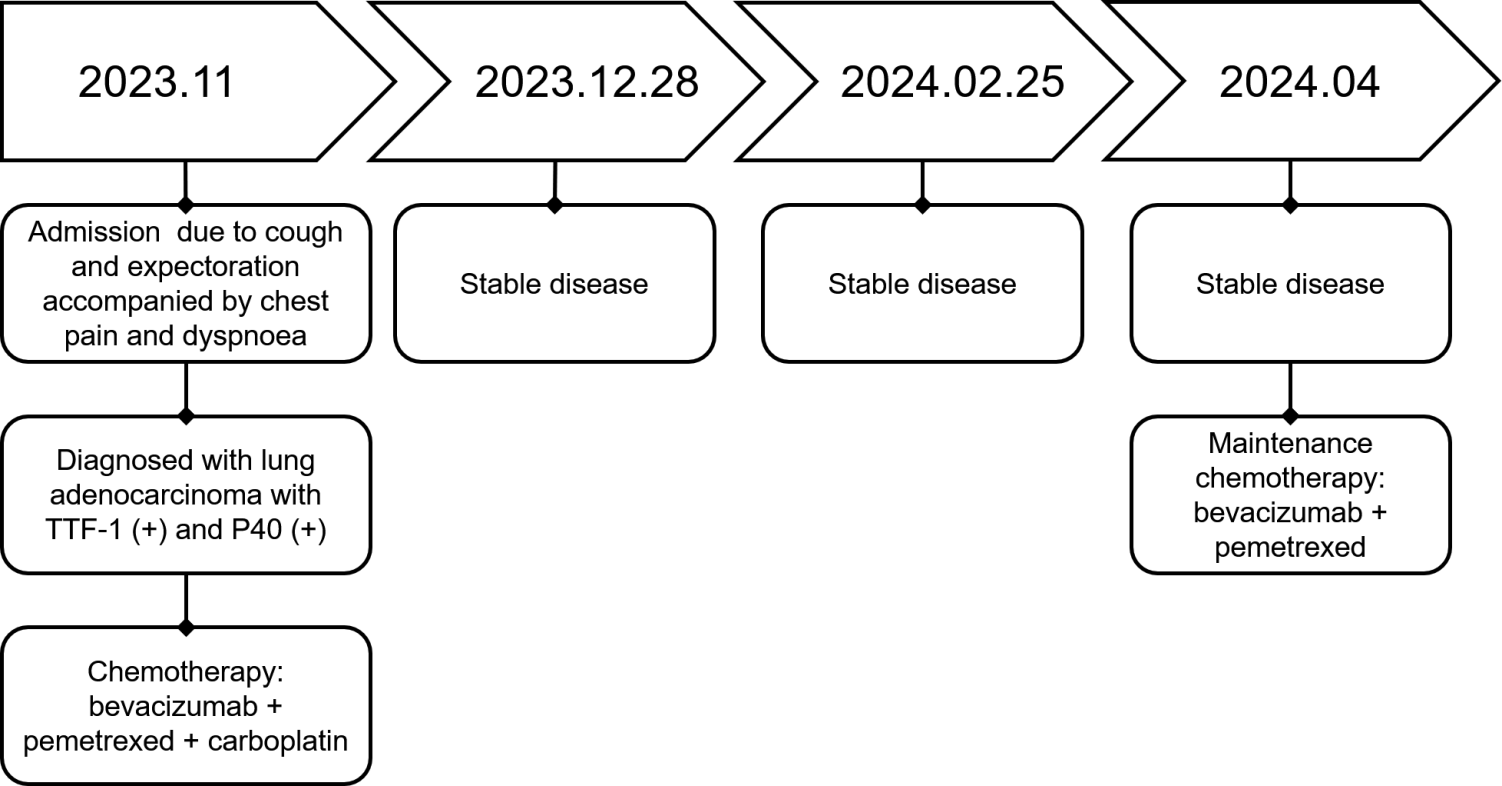
Course and treatment of patient.

A 53-year-old male patient without a smoking history visited our hospital due to cough and expectoration accompanied by chest pain and dyspnea in November 2023. Physical examination upon admission showed bilateral thoracic symmetry, disappearance of respiratory sounds in the lower lobe of the right lung and minimal scattered wheezes in the right and left lungs. However, palpable superficial lymph nodes or swelling was not observed. Plain and contrast-enhanced chest and upper abdominal computed tomography scans revealed perihilar lesions in the upper lobe of the right lung with distal obstructive pneumonia, invasion into the adjacent structures of the mediastinum, multiple metastases in the right and left lungs, possible lymph node metastasis in the mediastinum and right hilar lung, pleural effusion and a slightly thickened pleura in the right lung and multiple metastatic lesions in the liver (Fig. 1A). Whole-body bone imaging revealed abnormal increases in the radioactivity of the skull, sternum, cervical, thoracic, lumbar and sacral vertebral bodies, multiple ribs on both sides, scapula, pelvis and lower limbs, which indicated extensive bone metastasis (Fig. 1B). Thoracic puncture and drainage were performed for dyspnea. According to the results of exfoliative cytology of drainage fluid and immunohistochemistry [CK (AE1/AE3) (+), CK56 (−), CD68 (−), CD45 (LCA) (−), TTF-1 (+), p40 (+), D2-40 (−), and ki-67 (50%+)], adenocarcinoma of lung origin was considered (Fig. 2A–F). Second-generation sequencing showed ERBB2 (EXON20.p.Y772-A775dup) and TP53 (EXON5.c.404G-Tp.C135F) mutations (Fig. 3). The patient received 6 cycles of chemotherapy regimen comprising bevacizumab + pemetrexed + carboplatin. On December 28, 2023, and February 25, 2024, the patient was assessed to evaluate treatment efficacy, and he then achieved stable disease (SD) (Fig. 4A and B). At present, the patient is receiving a maintenance chemotherapy regimen comprising bevacizumab + pemetrexed and is in good condition. The course and treatment flow chart of patients (Fig. 5).

## 3. Discussion

Histopathological diagnosis plays an important role in lung cancer diagnosis. However, there are differences between morphological and immunohistochemical diagnoses. Further, the direction of differentiation cannot be determined based on the morphology of poorly differentiated malignant tumors. Consequently, the WHO (2015) made significant changes to the classification of tumors in the lung, pleura, thymus, and heart. Moreover, it showed that tumor classification must be based on immunohistochemistry results. TTF-1 and NapsinA are the commonly used immunohistochemical indicators of lung adenocarcinoma. Meanwhile, p63, ΔNp63/p40 and CK5/6 are the immunohistochemical indicators of squamous cell carcinoma. In non-small-cell carcinomas, whose direction of differentiation cannot be identified on morphological examination, tumors that test positive for TTF-1 should be reclassified as NSCLC, inclined towards adenocarcinomas, regardless of the co-expression of squamous cell carcinoma. Due to the high specificity of p40, its diffuse positivity strongly indicates the differentiation of squamous cell carcinoma. Currently, there are no specific guidelines for the diagnostic criteria of patients with NSCLC who present with TTF-1/p40 co-expression.

In this case, the patient experienced dyspnea caused by pleural effusion. To alleviate his dyspnea, thoracic puncture and drainage were performed. Hemorrhagic pleural effusion was drained. Then, exfoliative cytology of the pleural effusion and immunohistochemistry were conducted. Results showed diffuse TTF-1/p40 co-expression in the cancer cells, which indicated that they exhibited differentiation of both glandular and squamous cells simultaneously. Hence, the diagnosis of such tumors (poorly differentiated lung adenocarcinoma, NSCLC with immunophenotypes of adenocarcinoma and squamous cell carcinoma, or NSCLC-NOSS) remains unclear. The Association for the Study of Lung Cancer/American Thoracic Society/European Respiratory Society international multidisciplinary classification of small-specimen lung cancer shows that NSCLC should be further classified using small-biopsy specimens collected from patients with poorly differentiated cancers. Further, terms such as NSCLC and NSCLC-NOSS should be used as minimally as possible. For these types of lung cancer, classification should be based on light microscopy or special staining. In relation to this, we retrospectively analyzed similar cases reported in previous literature. As of January 2024, 23 cases of diffuse TTF-1/p40 co-expression have been reported in PubMed,^[4–12]^ as shown in Table 1.

**Table 1:** Clinical, pathological, and molecular characteristics of the reviewed cohort.

Reference	Age	Sex	Smoking history (Y/N)	Neoplasm diameter (cm)	Neoplasm localization	Metastasis localization	Therapy	Follow-up (months)	Molecular alterations
Pelosi (2015)^[5]^	77	Male	Y	8.5	Left (hilar)	Pleural effusion	None	DOD (1)	KRAS K117N, TP53 V272G, FGFR1 amplification
Hayashi (2018)^[6]^	73	Male	Y	19	LUL	No	Surgery	NA	ATK phosphorylation, PTEN H123D, TP53 V272L
Spinelli (2019)^[4]^	51	Male	Y	3.1	RUL	Brain, adrenal gland, mediastinal LN	RT + CT	DOD (3)	WT
Pelosi (2021)^[7]^	62	Female	Y	4.5	Right	Brain, Intrapulmonary, mediastinal LN	CT + Gefitinb	DOD (48)	EGFR E746_A750del, TP53 E224D, RAD51B P365R, CCND3 S259A
	62	Male	Y	4.7	LLL	Mediastinal LN, liver	CT + Pembrolizumab	DOD (3)	NF1 R1769
Tan (2021)^[8]^	85	Male	Y	5.7	Right	Mediastinal LN	CT + Pemetrexed	AWD (NA)	ALK
Cai (2022)^[9]^	38	Male	N	4.5	Right (mediastinum)	Bilateral intrapulmonary, bone, mediastinal LN	Carboplatin + Pemetrexed	NA	EmaleL4::ALK, PIK3CA S553T
Savari (2022)^[10]^	26	Female	N	5.2	LLL, central	No	Surgery	AWD (17)	DEK:AFF2, PATCH1 H1316Y, FGFR3 K403, BLM T1015I, SHH R244S, OLIG2 A227_A223del, WNK1 I726
Savari (2023)^[11]^	77	Male	Y	4.4	Peripheral	Brain, liver	NA	DOD (10,5)	TP53 and CDKN2A missense mutation, FGFR1 amplification
	68	Female	Y	4.2	Peripheral	Brain	NA	AWD (55)	TP53 missense mutation, KRAS G12C, FGFR1 and MYC amplification
	84	Female	NA	2.3	NA	No	Surgery	NA	TP53 splice site mutation, FGFR1, MYC, NKX2-1 and AKT1 amplification
	80	Male	Y	6.4	Central	NA	NA	DOC (22,6)	NA
	67	Female	NA	6	Peripheral	No	Surgery	NA	NA
	90	Female	Y	3	Peripheral	Intrapulmonary, supraclavicular LN	Surgery	DOD (51,3)	TP53 missense mutation, FGFR1, MYC, NKX2-1 and AKT1 amplification
	69	Male	Y	2.2	Peripheral	Liver, adrenal gland, bone, pancreas, stomach	NA	DOD (11,8)	NA
	65	Male	Y	3.4	Peripheral	No	NA	AWD (34,5)	TP53 splice site mutation, CDKN2A missense mutation, KRAS G12C
	79	Female	NA	NA	NA	NA	NA	NA	NA
	86	Male	NA	2.2	NA	NA	NA	NA	NA
	73	Female	NA	5.2	NA	NA	NA	NA	NA
	79	Male	Y	1.3	Central	No	Surgery	NA	TP53 missense mutation
	74	Male	Y	1.3	Peripheral	Mediastinal LN, pleura	NA	AWD (1,3)	TP53 and CDKN2A truncating mutation, FGFR1 amplification
	94	Female	N	1.9	Peripheral	No	RT	AWD (12)	EGFR N771_H773dup
Malearando (2024)^[12]^	64	Female	Y	5	RUL	Mediastinal LN, bone, adrenal gland, liver, brain	RT	DOD (3)	KRAS G12D, MET 14 exon skipping
Present case	52	Male	Y	6	Right	Mediastinal LN, pleura	Carboplatin + pemetrexed + bevacizumab	AWD	TP53 mutation, ERBB2 mutation

AWD = alive with disease, CT = chemotherapy, DOC = dead of other causes, DOD = dead of disease, LLL = left lower lobe, LN = lymph nodes, LUL = left upper lobe, NA = not available, RUL = right upper lobe, RT = radiotherapy, VUS = variant of unknown significance, WT = wild type.

The treatment options for squamous cell carcinoma and non-squamous cell carcinoma in NSCLC differ. Hence, an accurate diagnosis can affect the clinical selection of further treatment options. In this case, the patient presented with advanced-stage NSCLC, with diffuse co-expression of TTF-1/p40 in the same cell population. Currently, this type of NSCLC has not been clearly classified. The National Comprehensive Cancer Network, European Society for Medical Oncology and Chinese Society of Clinical Oncology guidelines recommend that patients with this type of lung cancer should initially undergo molecular genetic testing to identify potential therapeutic targets. ERBB2 (EXON20.p.Y772-A775dup) and TP53 (EXON5.c.404G-Tp.C135F) mutations were detected. However, there are no currently available drugs targeting TP53 mutations in the market domestically or internationally. Targeted drugs such as trastuzumab, pertuzumab, pyrotinib, and disitamab vedotin have been listed in China. However, none of them are approved for the treatment of patients with advanced-stage lung cancer who presented with ERBB2 amplification. Therefore, the chemotherapeutic regimen comprising bevacizumab (15 mg/kg, d1) + pemetrexed (500 mg/m^2^, d1) + carboplatin (AUC = 5, d1, q21) was selected. Moreover, a chest computed tomography scan was performed every 2 cycles to evaluate treatment efficacy. The patient achieved SD. After 6 cycles of chemotherapy, the patient’s condition remained stable, and he is currently undergoing regular follow-up.

According to the patient’s diagnosis and treatment, the following points should be explored in depth. First, this type of lung cancer is not clearly classified by the WHO. According to Spinelli et al, it can be a novel type of lung cancer and cannot be further classified as adenocarcinoma or adenosquamous carcinoma. Cabibi hypothesized that TTF-1/p40 co-expression in poorly differentiated NSCLC can originate from the basal-reserve cells of the terminal respiratory unit. These NSCLCs are characterized by various histogenetic lineages, higher clinical invasiveness and basal-like tumors resembling the breast. However, the existing case reports and small-scale single-center retrospective analyses cannot support the accurate classification of this disease. In the future, further large-scale multi-center studies should be performed to validate their histopathological and genetic features and histogenetic association with basal cells of the terminal respiratory unit and to further validate whether they constitute the true subtype of lung adenocarcinomas. Second, the treatment plan of this patient was selected based on the principles of the NCCNC, ESMO, and CSCO guidelines. The patient initially underwent second-generation sequencing, which revealed ERBB2 amplification and TP53 mutation. According to the existing literature in PubMed, the incidence rate of TP53 mutations in patients with this type of lung cancer is as high as 61.1% (11/18). TP53 mutations may be significant in the diagnosis and prognostic prediction of this type of lung cancer. In addition to the high frequency of TP53 mutations, patients with this type of cancer often present with other mutations such as K-ras mutations, MYC amplification, MET exon 14 skipping mutations and CDKN2 mutations. The molecular features of this type of disease are extremely complex, and an in-depth analysis and summary of its molecular features are significantly important in improving the understanding of this disease and exploring effective treatment plans. In this case, based on second-generation sequencing, the patient presented with not only TP53 mutation but also ERBB2 amplification, which has not been reported in previous literature as a therapeutic target. Although there are corresponding targeted drugs available in the market, they cannot be applied because of their lack of approved indications for NSCLC. Nevertheless, these drugs can be possible options for second-line treatment after disease progression in the later stage. Finally, the current first-line treatment for driver gene-negative advanced-stage NSCLC is still a platinum-based two-drug regimen combined with immune checkpoint inhibitors (KEYNOTE-189,^[13]^ CAMEL III,^[14]^ ORIENT-11,^[15]^ and GEMSTONE-302^[16]^). Considering that the TPS of this patient was 0, bevacizumab combined with a platinum-based two-drug regimen was selected based on the BEYOND research results.^[17]^ The patient achieved temporary SD. However, the clinical benefits of bevacizumab + PDL-1 inhibitors + platinum-containing two-drug regimen, based on the success of the IMPOWER150 study on NSCLC,^[18]^ are not yet elucidated. Further, the superiority of first-line immune checkpoint inhibitors combined with platinum-containing two-drug regimens superior remains unknown. Therefore, further randomized controlled trials must be performed to confirm these notions. In addition, this study focuses on NSCLC patients with clinically confirmed TTF-1/P40 co-expression, retrospectively analyzing 22 similar cases that were previously reported. Given the rarity of such lung cancer patients in clinical practice, the analysis and summary of these 23 cases may not be sufficient to fully describe the overall diagnostic and treatment status of this patient group. In the future, enhancing collaboration among medical institutions and establishing a tissue sample bank and a clinical research database for this patient cohort will be a highly challenging yet meaningful endeavor.

Current reports have shown that poorly differentiated NSCLC with TTF-1/p40 co-expression is associated with factors including male sex, older age, smoking history, poor histological differentiation, dual immunohistochemical features of adenocarcinoma and squamous cell carcinoma, lack of typical genetic changes in adenocarcinoma or squamous cell carcinoma, rapid clinical progression, and poor prognosis. Unlike patients in previous reports, the patient in this case had no smoking history. However, he presented with dual immunohistochemical features and ERBB2 mutation.

## 4. Conclusion

In conclusion, poorly differentiated NSCLC with TTF-1/p40 co-expression can be a novel type of lung cancer. Due to the limited number of known cases, more research must be performed to further analyze its features and molecular mechanisms and to confirm its various morphological features and treatment options.

## Author contributions

**Conceptualization:** Jiachun Sun, Dengkui Wang, Xinyang Li.

**Data curation:** Tingting Wei, Bo Sun, Haolin Shi.

**Investigation:** Jiachun Sun, Jingxiang Su, Hongyan Liu.

**Writing – original draft:** Jiachun Sun, Xinyang Li.

**Writing – review & editing:** Jiachun Sun, Haolin Shi, Xinyang Li.

## References

[1] Kratzer TB Bandi P Freedman ND. Lung cancer statistics, 2023. Cancer. 2024;130:1330–48. 38279776 10.1002/cncr.35128

[2] Canberk S Field A Bubendorf L. A brief review of the who reporting system for lung cytopathology. J Am Soc Cytopathol. 2023;12:251–7. 37156705 10.1016/j.jasc.2023.04.002

[3] Pelosi G Fabbri A Bianchi F. Δnp63 (p40) and thyroid transcription factor-1 immunoreactivity on small biopsies or cellblocks for typing non-small cell lung cancer: a novel two-hit, sparing-material approach. J Thorac Oncol. 2012;7:281–90. 22071786 10.1097/JTO.0b013e31823815d3

[4] Spinelli M Khorshad J Viola P. When tumor doesn’t read textbook. Third case of Ttf1 and p40 co-expression in the same tumour cells in a non-small cell carcinoma. A potential new entity to consider? Pathologica. 2019;111:58–61. 31388196 10.32074/1591-951X-12-19 PMC8186011

[5] Pelosi G Fabbri A Tamborini E. Challenging lung carcinoma with coexistent Δnp63/p40 and thyroid transcription factor-1 labeling within the same individual tumor cells. J Thorac Oncol. 2015;10:1500–2. 26398824 10.1097/JTO.0000000000000553

[6] Hayashi T Takamochi K Yanai Y. Non-small cell lung carcinoma with diffuse coexpression of thyroid transcription factor-1 and Δnp63/p40. Hum Pathol. 2018;78:177–81. 29410129 10.1016/j.humpath.2018.01.023

[7] Pelosi G Bulloni M Vescio M. Coexpression of Δnp63/p40 and Ttf1 within most of the same individual cells identifies life-threatening NSCLC featuring squamous and glandular biphenotypic differentiation: clinicopathologic correlations. JTO Clin Res Rep. 2021;2:100222. 34746884 10.1016/j.jtocrr.2021.100222 PMC8551500

[8] Tan Y Lu X Li Y. ALK-positive pulmonary adenocarcinoma with signet ring features (PASRF) and polygonal cell morphology simultaneously co-expressing Ttf-1/P63/P40: a case report. Transl Cancer Res. 2021;10:3864–9. 35116685 10.21037/tcr-21-335 PMC8797292

[9] Cai Y Liu H Chen X Yang J Huang B. p40 and TTF-1 double-expressing non-small cell lung cancer with EML4-ALK and PIK3CA gene mutations: a case report and review of the literature. Oncol Lett. 2023;25:59. 36644157 10.3892/ol.2022.13645 PMC9827448

[10] Savari O Chang JC Bishop JA Sakthivel MK Askin FB Rekhtman N. First report of thoracic carcinoma with DEK::AFF2 rearrangement: a case report. J Thorac Oncol. 2022;17:1050–3. 35773081 10.1016/j.jtho.2022.05.009 PMC9357138

[11] Savari O Febres-Aldana C Chang JC. Non-small cell lung carcinomas with diffuse coexpression of TTF1 and p40: clinicopathological and genomic features of 14 rare biphenotypic tumours. Histopathology. 2023;82:242–53. 36130728 10.1111/his.14801 PMC10501689

[12] Marando A Zagni M Negrelli M. Biphenotypic lung carcinoma with coexpression of TTF-1 and ΔNP63/p40 within most of the same individual cells: a further case confirming poor prognosis and a review of literature. Pathologica. 2024;116:13–21. 38482671 10.32074/1591-951X-957 PMC10938280

[13] Garassino MC Gadgeel S Speranza G. Pembrolizumab plus pemetrexed and platinum in nonsquamous non-small-cell lung cancer: 5-year outcomes from the phase 3 keynote-189 study. J Clin Oncol. 2023;41:1992–8. 36809080 10.1200/JCO.22.01989 PMC10082311

[14] Ren S Chen J Xu X. Camrelizumab plus carboplatin and paclitaxel as first-line treatment for advanced squamous NSCLC (Camel-Sq): a phase 3 trial. J Thorac Oncol. 2022;17:544–57. 34923163 10.1016/j.jtho.2021.11.018

[15] Zhang L Wang Z Fang J. Final overall survival data of sintilimab plus pemetrexed and platinum as first-line treatment for locally advanced or metastatic nonsquamous NSCLC in the phase 3 orient-11 study. Lung Cancer. 2022;171:56–60. 35917647 10.1016/j.lungcan.2022.07.013

[16] Zhou C Wang Z Sun Y. Sugemalimab versus placebo, in combination with platinum-based chemotherapy, as first-line treatment of metastatic non-small-cell lung cancer (Gemstone-302): interim and final analyses of a double-blind, randomised, phase 3 clinical trial. Lancet Oncol. 2022;23:220–33. 35038432 10.1016/S1470-2045(21)00650-1

[17] Zhou C Wu YL Chen G. Beyond: a randomized, double-blind, placebo-controlled, multicenter, phase iii study of first-line carboplatin/paclitaxel plus bevacizumab or placebo in Chinese patients with advanced or recurrent nonsquamous non-small-cell lung cancer. J Clin Oncol. 2015;33:2197–204. 26014294 10.1200/JCO.2014.59.4424

[18] Socinski MA Nishio M Jotte RM. Impower150 final overall survival analyses for atezolizumab plus bevacizumab and chemotherapy in first-line metastatic nonsquamous NSCLC. J Thorac Oncol. 2021;16:1909–24. 34311108 10.1016/j.jtho.2021.07.009

